# An exploration of barriers and facilitators to older adults’ participation in higher impact physical activity and bone health: a qualitative study

**DOI:** 10.1007/s00198-015-3376-7

**Published:** 2015-11-10

**Authors:** B. A. J. Simmonds, K. J. Hannam, K. R. Fox, J. H. Tobias

**Affiliations:** 1 Musculoskeletal Research Unit, Learning and Research Building, The University of Bristol, Level 1, Southmead Hospital, Bristol, BS10 5NB UK; 2 Centre for Exercise Nutrition and Health Sciences, School for Policy Studies, The University of Bristol, 8 Priory Road, Bristol, BS8 1TZ UK

**Keywords:** Bone health, High impact, Older adults, Physical activity, Qualitative

## Abstract

**Summary:**

This qualitative study explored the acceptability of high-impact physical activity for increasing bone strength in later life. Thematic analysis established the barriers and facilitators to this physical activity. They prioritised joint over skeletal health, of which they had little concept. Interventions need to clearly communicate the rationale and benefits.

**Introduction:**

The aim of this study was to explore the acceptability of doing high-impact physical activity in later life.

**Methods:**

This qualitative study was embedded within a large-scale observational study and was designed to address specific objectives and feed into a subsequent intervention. Five focus groups with physically active men and women (over 50 years) were used to develop an interview topic guide to explore the acceptability of high-impact physical activity in older men and women (over 65 years) in South West England. A total of 28 semi-structured interviews with 31 participants were then conducted and transcripts analysed thematically.

**Results:**

Three main barriers emerged: conceptualising bone, damage to joints and falling/safety concerns. Two main facilitators were also identified: the need to understand clear tangible benefits and incorporation of activity into everyday habits. Older adults were interested how high-impact physical activity would help to maintain their mobility, independence or social relationships. Some participants wanted tangible feedback from accelerometers, health care professionals and/or bone scans in order to develop a more intimate knowledge of their bone health.

**Conclusions:**

Interventions incorporating high-impact physical activity for older adults need to communicate how this activity can impact more broadly on health and lives; that physical activity will be safe, beneficial and not damaging to their joints will need to be clearly conveyed. Ways in which high-impact physical activity can be habitualised into everyday activities, be fun and interactive may help facilitate longer term adoption.

## Introduction

In 2000, 66 % of fractures in women and 30 % of fractures in men were likely to be osteoporotic [1]. Fracturing bones in later life has a significant impact on health and wellbeing [2]. Hip fractures, in particular, lead to a decline in functional ability [3] and have a significant impact on morbidity and quality of life [4, 5]. Mortality rates amongst those over 50s after being hospitalised due to sustaining a hip fracture are high, with 36 % of men and 21 % of women dying within 1 year [5]. Having a fracture or fall can be an epiphanal moment in an older person’s life, when a border is crossed from independence to decline [6].

The evidence for the benefits of physical activity in later life in terms of physical, social and psychological wellbeing, lower incidence of emergency hospital admissions and use of medical prescriptions is growing [7–9]. Furthermore, evidence that physical activity can improve bone strength as assessed by measures such as bone mineral density (BMD) is also increasing (lesser evidence exists for reducing fractures) [10–18]. For example, a meta-analysis of studies investigating the effect of impact exercise on postmenopausal bone loss concluded that brief high-impact physical activity can increase bone mineral density (BMD) at the hip and spine in older women [15]. Structured exercise programmes which include high-impact exercise were found to be beneficial for bone strength, as well as more home-based physical activities such as jogging and stair climbing [15].

As an example of a home-based high-impact exercise intervention, a 12-month hopping intervention was recently found to increase BMD at the femoral neck in healthy community-dwelling older men (average age 61 years) [11]. The progressive exercise intervention culminated with five sets of 10 multi-directional hops every day [11]. Each set of hops was interspersed with a 10-s rest, and the effect was enhanced bone adaptation [19, 20]. Initial supervised sessions took place in a group setting once a week for a month and then once a month for the following 3 months; men hopped in their own homes for the remainder of the intervention [11]. The acceptability of this intervention appeared to be good with adherence to the intervention being 91 %, with only three men withdrawing, citing injuries caused by the intervention. However, the feasibility and acceptability of this intervention with older frailer adults remain to be established.

The barriers to physical activity in later life are already well documented and include the ageing body, practical issues (e.g. transport, facilities and cost) and socio-cultural identities [6]. There are also studies documenting experiences of arthritis pain, fatigue and stiffness [21, 22], but to the authors’ knowledge, no previously published qualitative study has explored the acceptability to older adults of participation in a high-impact exercise intervention specifically designed to improve bone health. Literature examining patient understanding of fracturing and osteoporosis found that people at risk of osteoporotic fracture found it difficult to relate to their bone strength and the term ‘fragility fracture’ [23]. Participants in another study reported uncertainty with the level of impact their ‘inside’ bones could endure when subjected to ‘outside’ realities of the social world; they also received contradictory health messages about physical activity and damage to joints [24]. Older people’s perceptions of falls prevention interventions have also been explored in qualitative studies, with the maintenance of social relationships and independence being given high priority [25, 26].

Given the importance of high-impact physical activity in improving bone health in older adults [11, 15] and the limited existing research regarding understanding and perceptions of higher impact exercise, in this qualitative study, we aimed to explore the acceptability of carrying out high-impact physical activity in later life, with a focus on potential barriers to and facilitators for jumping in individuals’ home environment (inside/outside). This was to avoid capturing the barriers and facilitators to older people attending physical activity sessions not in their home environment, which are already well documented [6].

## Methods

**Fig. 1:**
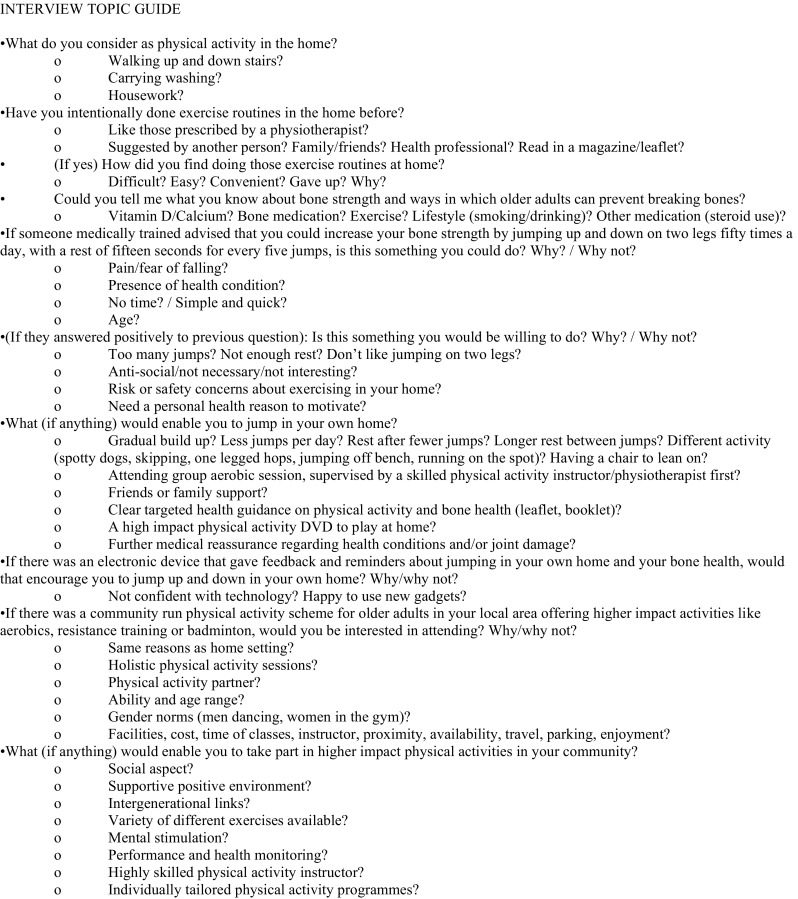
Interview topic guide

This qualitative study was embedded within a large-scale observational study and was designed to address specific objectives and feed into a subsequent intervention. The fieldwork took place in Bristol, in South West England, between November 2014 and March 2015. Five focus groups were initially carried out with female (*n* = 29) and male (*n* = 7) participants over 50 years who regularly undertook high-impact exercise. The findings were then used to inform the development of a topic guide for semi-structured interviews with older men and women (over 65 years) drawn from the general public (Fig. 1), on which the present results are based.

Interview participants were purposively recruited from a sample of ethnically and socially diverse older adults (over 65 years) ranging in functional mobility levels who were members of community groups of older adults in South West England. Gatekeepers of these groups were identified through networking with umbrella organisations, attending and presenting at a local community health and wellbeing event for older people and presenting the research on a radio show focusing on health and wellbeing [27]. Initial contact was made with the gatekeepers of a diverse range of groups working with older people to explain the study and ask permission to attend the group to talk to their members about the research. The researcher (BS) attended the community groups run for older adults, explained the research to the members and took the contact details of any interested members. Participant information packs were sent by mail, and interviews were arranged if the consent form and reply slips were received.

In total, 63 older adults were sent participant information packs, and 31 consented and took part in an interview. Three interviews took place with two interviewees; therefore, in total, 28 interviews were completed. Interviews took place in mutually convenient locations for participants (i.e. in their own homes, in community buildings or cafes). Semi-structured interviews were chosen because it enables the focus of the interview to be maintained, whilst allowing flexibility for the priorities of older adults themselves to be expressed [28]. Data collection and analysis was an iterative process, and data collection ceased when saturation was reached and no new themes emerged [28]. Before interviews took place, a short standardised questionnaire was completed to obtain demographic information and mobility level information (age, gender, ethnicity, self-reported walking speed and walking aid use). An indication of socio-economic status is presented in Table 1 by converting postcodes into index of multiple deprivation (IMD) quartiles.

**Table 1:** Demographic characteristics of interview participants

Characteristic categories	*N* (%)
Gender	
Female	23 (74)
Male	8 (26)
Ethnicity	
White	21 (68)
Black	5 (16)
Asian	5 (16)
Age	
65–75	15 (48)
75–88	16 (52)
Self-reported walking speed	
Easy pace/slow	16 (52)
Normal/brisk/fast	15 (48)
Use of walking aids	
Zimmer frame/trolley/walking stick	11 (35)
None	20 (65)
IMD quartile^a^	
1 (most deprived)	6 (19)
2	16 (52)
3	7 (23)
4 (least deprived)	2 (6)

^a^Index of multiple deprivation quartiles based on English national standards

The topics raised in the semi-structured interviews included asking older adults about their current physical activity and hobbies, their understanding of bone health, jumping in their own home environment, if they would consider participating in a group exercise setting with higher impact physical activities, if not why not and what would encourage or facilitate their participation in higher impact physical activity (if anything) (Fig. 1).

The interview transcripts were thematically coded and organised into a framework [29] using NVivo (version 10). In step 1, all the data were coded into meaningful text segments. In step 2, themes were then developed and refined into discrete categories. In step 3, themes were then arranged into hierarchies with basic themes under sub-themes and global themes such as ‘barriers’ or ‘facilitators’ at the top, forming a thematic framework. In step 4, each theme was described and the text segments explored in more detail. Themes were made explicit using empirical examples in step 5. In step 6, the themes were interpreted. To ensure dependability and credibility of analysis, six (20 %) of the interview transcripts were double coded by another qualitative researcher; an analysis meeting enabled the researchers to discuss any differences and come to a consensus [30]. An abductive analysis strategy was utilised where data were interpreted with existing theory, whilst allowing for original findings to be developed from the data [31]. Differences and similarities were explored within the thematic framework, examining gender, ethnicity and mobility. However, due to the small numbers in each group, extensive sub-group analysis was limited.

All participants gave informed consent to take part, before the interview commenced. In the interview, they were reminded of their right to withdraw, their confidentiality and that any quotes will be anonymised using pseudonyms. The study protocol was approved by the Faculty of Medicine and Dentistry Research Ethics Committee at the University of Bristol.

## Results

Participant characteristics are shown in Table 1. Of the 31 participants who consented and took part in an interview, 23 were women and 8 were men. Twenty-one participants were White British, five participants identified with an Asian ethnicity and five identified with a Black ethnicity. The average age of participants was 75 (range 65–88). Twelve participants lived alone as a result of widowhood or divorce. Twenty-two participants were from IMD quartiles 1 and 2 (less advantaged areas).

Four participants disclosed unprompted that they had fractured a bone. Five participants disclosed that they had had joint replacements at the knee, shoulder or hip, and 22 participants stated that they had experienced physiotherapy usually following an operation or injury. Sixteen participants identified themselves as having an ‘easy/slow’ walking speed; the remainder described their walking speed as ‘normal/brisk/fast’. Eleven participants stated that they used a walking aid (trolley/frame/walking stick), and the remainder stated that they did not use any form of walking aid.

Three main barriers emerged from the data: difficulty in conceptualising bone, concern over damage to joints and falling/safety concerns. Two main facilitators were identified: the need to understand clear tangible benefits and the need to incorporate any behavioural change such as increased high-impact physical activity into everyday habits.

### Barrier 1: conceptualising bone

Participants were first asked about their understandings of bone health. This revealed the confusion and difficulties conceptualising bone and subsequent fracture risk amongst this population. Participants reported that bones were difficult to relate to as they felt that they cannot be seen and rarely can they be felt. For example, Colin (aged 85) explained that he was told to watch out for pain in his bones with regard to another health condition that he has experienced. He stated:

I keep saying to myself now “This pain down my leg – is it the bone? What does bone pain feel like?” … I often wonder what it feels like. I tried to find out on the internet, but it didn’t say. They said there was a dull, nagging pain, but it’s not that… you can’t really feel them. You can feel the joints well enough.

Although Colin is not talking here about bone fracture pain, it highlights what other participants expressed about bone health, which is fracture risk is a theoretical concept and until you have experienced a fracture, there is no pain or warning signs. For instance, when Colin was asked whether he would need feedback from high-impact physical activity, he stated:

I’d just assume that they were strong enough not to break. If they did break I’d say “Well dammit, it didn’t work!” [laughs].

The lack of corporeal bone sensation until participants experience a fracture is a significant barrier to the conceptualisation of bone and fracture risk. Most participants when asked about their understandings of bone health talked about their joints, referring particularly to arthritis. Some felt pains in their joints on a daily basis. For example, Sally (aged 74) reported that she felt pain in her bones on a daily basis.

- INT: Can you just tell me what you understand about bone health?
- Sally: Oh my God, sometimes if I even move, or lay down, it’s too hard for me to move it, because I have got pain here, and over the fingers and going stuff, and making too much noise!
- INT: Where did you feel pain?
- Sally: Everywhere (laughs)!

Older adults mentioned that supplements such as cod liver oil, glucosamine, calcium and vitamin D were good for bone health. An excerpt from Miranda (aged 73) captures the confusion between bone and joint health in relation to the supplements she takes:

- INT: Can you just tell me what you understand about bone health and things that you can do to prevent your bones getting weak.
- Miranda: Well, I’ve always heard about brittle bones and I’ve started taking glucosamine.

Diet was thought to be important, and when asked about bone health, participants mentioned fruits, vegetables, dairy and protein as all being good for bones. Exercise was also mentioned, but this is likely to have been prompted by the topic of this study. However, the activities discussed (swimming, cycling and walking) suggest that understanding of the types of physical activity that may be good for strengthening bones is limited.

Participants’ general focus was on joint care rather than bone health, which could be linked to some already having had joint replacements and the advice given to avoid high-impact physical activity. The conceptualisation of bone health and fracture risk is central to determining the acceptability of higher impact physical activity. If individuals struggle to conceptualise and relate to their bone strength, they are unlikely to accept and commit to an intervention to promote bone strength.

### Barrier 2: damaging joints

The most frequently cited barrier to participating in higher impact physical activity was the damage this would cause to joints. For example, when Veronica (aged 88) and Rosy (aged 73) were asked whether they would consider doing some higher impact physical activity, they stated:

- Veronica: I’m not sure, but I’d rather not try it!
- Rosy: No, ‘cause you’ve got those false hips haven’t you? It’s your hips that would do it.

Other participants mentioned their joint replacements (hips and knees) as a reason why they would be hesitant to participate in higher impact physical activity. Janice (aged 72), for example, has been advised by a consultant who replaced her knee not to jump:

It’s since my surgery, or since my knees became bad, that I don’t run, and the consultant has advised me not to go running. He said, “Running for you, now, would be stupid.” That was how he put it [laughs] … I think the consultant is also worried about me damaging my knees. I think I’m very careful because I know it’s a prosthesis in there … I used to jump … but since my surgery, I do more low-impact exercise … because the knee replacement … it’s a major operation.

Due to the presence of other comorbidities, some older adults felt they would need medical advice from their general practitioner (GP) or other health professionals, like an osteopath or physiotherapist, before they attempted to jump. For example, when asked whether she would jump in her own home environment, Elaine (aged 86) replied:

I would do it if it was on the doctor’s recommendation, yes. If he thought I was capable of doing it … I wouldn’t undertake it unless I was advised. Or unless I confirmed it was safe to do it.

### Barrier 3: falling/safety/other concerns

Fear of falling, safety and other social-psychological concerns also acted as barriers to higher impact physical activity. Nine participants recounted previous falling experiences or admitted to previously having a fall; therefore, many were scared of subsequent falls, especially if they lived alone. Some participants wore alarms around their necks whilst in their home environment to help them if a fall occurred or they slept downstairs, as stairs were difficult to climb and posed a risk. However, most interviewees who had fallen in the past felt they would jump if they were holding onto the back of a chair and/or they were supervised by a specially trained instructor in a group setting.

- INT: If a doctor or a physio said to you, “We want you to jump up and down in your own home…”
- Betty (aged 82): As long as I’m not going to fall, I will do it [laughs].
- INT: Okay, yes, because if you were holding onto a chair – is it safety that you’re worried about?
- Betty: Yes.

Jumping was perceived to be an unusual (even humorous) activity that older adults had not participated in for a long time. Listening to their body and accepting that they could no longer do the activities they once did was a common narrative amongst this older population. Due to the unfamiliar nature of jumping, some older adults were concerned about muscle stiffness. Others felt that they could not jump or had balance and mobility problems. However, once holding onto a chair was suggested, most would consider building up jumping from a small number (e.g. three sets of 10 a day). Four people tried jumping whilst in the interview and it seemed acceptable to them. Four participants stated that they would not jump either in the home environment holding onto a chair or supervised in a group setting due to health and safety reasons. A further two participants stated they would not do any higher impact physical activity, either because they were already doing enough physical activity or because they did not think higher impact physical activity would be of benefit to them and would rather walk. Some gender-specific barriers to jumping included being body conscious and therefore feeling embarrassed about jumping, considering suitable bra support and having weak bladder control. Ethnic or cultural barriers were few. Doing physical activity in mixed-gender groups in Asian communities was a consideration however; this was not an issue for Afro-Caribbean and White British ethnic groups. A man with South Asian ethnicity had mobility problems because he declined a knee replacement, as it would impede kneeling to pray, and this now made physical activity difficult.

### Facilitator 1: clear tangible benefits

As described above, older people found it hard to conceptualise fracture risk; therefore, an important facilitator to taking part in higher impact physical activity was the communication of clear tangible benefits. Participants required a clear rationale as to why higher impact physical activity would be beneficial, particularly as it directly opposes advice and previous public health messages advising them to avoid high-impact physical activity to protect their joints. For instance, the following excerpt details Toby’s (aged 74) reaction when asked when he last jumped:

[Laugh] Last time I ever jumped? [Pause] I can’t really remember. Not recently anyway. Is this going to be the be all and end all? Is it? By jumping is it. What is it actually going to do?

This was fairly typical of many interviewees’ reactions to the suggestion that jumping would be of benefit to their health. Many older adults were interested in doing physical activities that maintained their independence in later life and protecting current social relationships. For example, when we asked why Molly (aged 66) continued to complete physiotherapy exercises for a shoulder injury, she explained the meaning and significance that doing them had on her life:

Because I can see that they are benefitting me … it’s just got to become part of routine … my neck this week, I can … feel that it’s a lot looser … to keep it mobile, I need to keep moving my head around … I don’t want to get into old, old age and not be able to do anything … Having to go in to a home and … be looked after …

Many interviewees said they would do anything beneficial to maintain their mobility in later life, including jumping. Participants were motivated by enjoyment and socialising, doing physical activity to music, intergenerational physical activity (with grandchildren) or devising challenges. Participants suggested if jumping was holistically beneficial (physically, socially and psychologically), this would facilitate sustainable participation. For instance, skipping was a familiar activity which most had participated in the past and was associated with fun and pleasure. Participants were also motivated by the potential to increase bone strength and potentially reduce fracture risk and the ways that could be objectively measured and fed back. The forms of feedback included an impact accelerometer (suggested by the researcher), a wall chart tracking jumping compliance, attending a supervised session and receiving feedback from a health professional, or having a before and after dual-energy X-ray absorptiometry (DXA) scan. For instance, Cindy (aged 66) clearly states here that if there were tangible results and feedback available to her, then she would probably jump on a regular basis.

It’s difficult to say because I think if it was visible … if … I don’t know. If an x-ray was taken of my legs and then after say 3 months of doing this jumping another x-ray was taken and I could see a difference if that’s what they said they were going to do then fine, I’d probably do it. But it’s like exercising, you know, once you’ve had the surgery you have to exercise to get better, get your joints moving. You can feel it. After a few days you can feel a slight difference so you know that what you’re doing is right. I don’t know if it would be the same for sort of … if there was a reason for it that I could see…

Participants were motivated by the observable benefits they could gain from doing this type of physical activity. The difficulty in conceptualising fracture risk provides justification for examining accessible methods of detecting benefits for older adults. Additionally, some older adults in this study were interested in finding out ways in which they could reduce their medication use and were interested in whether jumping to improve bone strength could reduce the need for medication. Other patients talked about the quantity of different medications for other comorbidities that they had been diagnosed with and the subsequent side effects, and were therefore keen to avoid any future pharmacological treatments.

### Facilitator 2: everyday habits

Interviewees reported that another facilitator would be to integrate higher impact physical activity into older adults’ activities of daily living or individual’s current physical activity routine. For instance, Lucy (aged 66) and Max (aged 67) stated:

- Lucy: I suppose if I thought it was doing me good … I mean it’s not that hard, is it, ten jumps every so often. That’s something you could do as you’re walking around, sort of thing. Every now and again I’ll have a go. Once you got into it, I suppose you could do it really just without thinking.
- Max: You’ll be jumping when you’re at the table [laughs].

Alternatively, others wanted the jumping to be flexible to their lives, their body and feelings that day. Some participants were extremely disciplined and already completed a battery of exercises every day, whereas others were more relaxed and would sporadically participate in physical activity depending on how they felt that day. For example, when asked about why Jenny (aged 84) found doing physiotherapy exercises at home difficult, she stated:

“Yes, I’ll do this at home”, and you get home and you think, “I’ll just do that”, and you might do it the next day as well and then the day… you say, “I’ll do it presently”, and you don’t get around to doing it.

Attending a supervised one-off (group) session demonstrating a variety of higher impact exercises was welcomed by most interviewees. Interviewees reported this would help with confidence, could sustain interest and be a potential socialising opportunity. For instance, Molly (aged 66) stated:

I think it would be good to go … and have it all explained, as once you know how things are working, that sort of helps you to look after yourself. Perhaps meet up sort of once a month just to keep you going really…

With regard to attending group sessions on a regular basis, older adults were interested, but only if convenient (time, transport, cost, timing and location) and of high quality (supervision, safe, tailoring and pitch).

## Discussion

In this study, when exploring the acceptability of doing high-impact physical activity, we found that older adults had difficulties with conceptualising their bones. Participants found it difficult to conceptualise abstract concepts like fracture risk as compared to joint pathology. Many older adults had already damaged their joints, had replacement surgery, felt pain every day and had been advised not to participate in high-impact physical activity. Participants highlighted a fear of falling and safety issues if they were to jump in their home environment; others would consider attending a higher impact physical activity group session with supervision. What would facilitate those who would be willing to jump in the home environment on an ongoing basis is to understand the benefits. Some would be particularly motivated by understanding how participating in high-impact exercise translates in terms of maintaining independence and current social relationships. Participants valued wider benefits from exercise, such as increased mobility and less medication use. In some cases, it would be helpful to have tangible feedback in terms of bone measures such as DXA scan readings. Another facilitator reported by older adults would be habitualisation of jumping in their activities of daily living.

Similar to the results of this study, Hurd Clarke et al. [32] found that older women diagnosed with osteoporosis focused on maintaining functional independence and being pain-free, to enable their participation in current social relationships. Other literature has found that older people attribute meaning to falls prevention interventions when related to maintaining independence and social relationships [25, 26]. Although women in Hurd Clarke et al.’s [32] study had a diagnosis of osteoporosis, none of the interviewees discussed the embodied experience of osteoporotic symptoms, such as feeling pain from fragility fractures. Bury’s [33] concept, ‘meaning as consequence’, could be useful in understanding how participants focused on the impact ill health could have on their lives, not their corporeal experience per se. Older people want to know how fragile bones will impact their lives in terms of their mobility, independence and social relationships, and are less concerned and conscious about how their bones will feel.

Nevertheless, a study exploring older adults’ experiences of fracturing bones had different findings. Clinicians used the term ‘fragility fracture’ and this resulted in patients disassociating themselves from having osteoporosis, and instead they experienced the fracture in an emotionally and physically traumatic manner [23]. Although this was not a finding of our study, participants felt disconnected from their bones. Similarly, in a work by Dalsgarrd Reventlow [24], interviewees explained the disconnect between the ‘inside’ risk of the bones breaking with the ‘outside’ reality of the social world around them, specifically the uncertainty related to the time until they break a bone and what force their bones could endure. Furthermore, older women’s fear of fracture increased whilst participating in physical activity [24]. These findings are similar to our findings that older adults had difficulty conceptualising the ‘inside’ of their bones and some were apprehensive about physical activity and risks associated with it. Furthermore, contradictory messages were being conveyed to older people regarding the protection of their joints whilst simultaneously encouraging them to participate in physical activity [24]. For instance, most participants with joint replacements, particularly at the knee, had been advised by their surgeons not to do physical activities involving impacts such as running. Therefore, considering advice to the contrary was a concern for our interviewees.

Participants wanted to know how doing high-impact physical activity will benefit them in terms of maintaining their mobility, independence and social relationships. Older adults found the conceptualisation of their bones and the theoretical concept of fracture risk difficult. This could explain why participants felt they wanted tangible feedback from impact accelerometers, feedback from health care professionals or DXA scans; they need to access the ‘inside’ and develop an understanding of their bone health. Giving information on bone health in terms of DXA scan results can be challenging, as people tend to underestimate their fracture risk and interpret a lack of information on their bone health as good news [34–36]. Nevertheless, the rationale and benefits to higher impact jumping in terms of preventing fracture and maintaining independence and social relationships need to be communicated, alongside the fun elements of jumping intergenerationally or when habitualised into their daily lives. A study has found that participating in higher impact physical activity could produce similar benefits to bone health medication [37]. Therefore, participants in this study were interested in jumping as opposed to taking bone medications, which could have a meaningful impact on an individual’s lives.

A strength of this study was the diversity of the sample, which included different ethnic groups, a range of ages, participants from less advantaged socio-economic groups and mobility levels. Physical activity studies are susceptible to attracting middle-class White British participants who are already regularly participating in physical activity [38]. Furthermore, by employing an abductive analytical approach, original findings emerged that might otherwise have been overlooked [31]. For example, semi-structured interviews enabled the collection of rich data whereby participants could express their priorities, and allowed us to present insights into their understandings of bone health and higher impact physical activity. Another strength was using five focus groups with already active older adults to develop a topic guide for a more general population, as some barriers and facilitators were already highlighted and could be explored thoroughly with the interviewees. The researcher built rapport when attending older people community groups, making them relaxed in her presence and willing to take part. A limitation of the study was the small sample size which did not allow for any sub-group comparative analysis. Finally, older adults from South West England self-selected to participate in this study; thus, the findings may not be representative of all UK older adults.

In summary, older adults who participated in this study had difficultly conceptualising their bones, insomuch as they had difficulty relating to the ‘inside’ and how this related to the ‘outside’ social world. Participants were interested in ‘meaning as consequence’ vis-à-vis how participating in higher impact physical activity can maintain their mobility, independence and social relationships. Therefore, interventions incorporating higher impact physical activity, such as jumping in their home environment, need to include careful communication strategies that help participants understand how this activity can impact meaningfully on their lives. Furthermore, the parameters within which the physical activity will be safe, beneficial and not damaging to their joints will need to be clearly conveyed. Finally, ways in which jumping can be habitualised into everyday activities and or can be a fun interactive (intergenerational) activity may help facilitate adoption of this type of movement in the longer term.
